# Malnutrition, Eating Habits, Food Consumption, and Risk Factors of Malnutrition among Students at the University of Maroua, Cameroon

**DOI:** 10.1155/2022/1431743

**Published:** 2022-08-08

**Authors:** Francoise Raissa Ntentie, Mary-Ann Angie Mbong, Boris Ronald Tonou Tchuente, Christine Fernande Biyegue Nyangono, Maxwell Wandji Nguedjo, Cedric Bissal, Palouma Souavourbe, Franck Avom-Me Mbida, Julius Enyong Oben

**Affiliations:** ^1^Department of Earth and Life Sciences, Higher Teachers' Training College, University of Maroua, Maroua, Cameroon; ^2^Laboratory of Nutrition and Nutritional Biochemistry, Department of Biochemistry, University of Yaounde 1, Yaounde, Cameroon; ^3^Centre for Food and Nutrition Research, Institute of Medical Research and Medicinal Plants Study, Ministry of Scientific Research and Innovation, Yaounde, Cameroon; ^4^Advanced Teacher Training College for Technical Education, University of Douala, Douala, Cameroon

## Abstract

**Methods:**

Three hundred and thirty students of both sexes, aged between 17 and 35 years old, were recruited from January to February 2018. Anthropometric parameters (weight, height, waist and hip circumference, and BMI) were measured, and an eating and lifestyle questionnaire was administered to each participant, as well as a 24-hour dietary recall.

**Results:**

The mean age of participants was 23.95 ± 3.67 years and BMI was 22.19 ± 2.78 kg/m^2^. Approximately 6.1% were underweight, 12.1% overweight, and 0.9% obese, and all age groups were affected. Concerning eating habits, low protein, fruit, and vegetable consumption were recorded among students. Cereals were the main source of carbohydrates consumed. Besides, 6% of them had a low dietary intake, 21% had a poorly diversified diet, and 2% were highly food insecure. The students' daily macronutrient intake was within the recommended reference values, except for carbohydrates, which exceeded and represented 62.69± 13.84% of daily energy intake. Also, only 32.2% of respondents had adequate energy intake. A poorly diversified diet was associated with a high incidence of overweight.

**Conclusions:**

Both forms of malnutrition are indeed present among the students of the University of Maroua, and nutritional education for this young segment of the population will be essential to prevent complications associated with malnutrition in the working life.

## 1. Introduction

Changes in people's diets, habits, and lifestyles observed in recent decades have contributed to an increase in malnutrition and diet-related chronic diseases [1]. According to Blössner and De Onis [2], malnutrition is a term that encompasses two situations: undernutrition due to a lack of essential nutrients caused by a poverty-related lack of food availability and overnutrition resulting from an energy imbalance between intake and expenditure in favor of intake. Undernourishment leads to a decrease in the immune defense system making the subject vulnerable to deficiency and opportunistic diseases such as malaria, diarrhea, typhoid, etc. The number of people affected by malnutrition worldwide is estimated at one billion each year; statistics vary from one geographical area to another [3]. Also, overnutrition exposes people to noncommunicable diseases such as cardiovascular disease, diabetes, hypertension, and cancer, whose prevalence increases exponentially worldwide, and sub-Saharan Africa is not spared [4]. The most effective strategy to fight these noncommunicable diseases is to prevent risk factors and their determinants at an early stage of life. Overweight and underweight in early adulthood affect health in old age, increasing the risk of morbidity and mortality. Therefore, there is a need to focus on the young population, particularly those of the academic community who are very sensitive to the nutritional transition. When these young adults move from secondary school to independent higher education or university, they become responsible for their eating habits and often have little or no knowledge to guide their food choices [5]. This exposes them to poor eating habits that can affect their health once they start their active life [6]. Many studies have reported poor eating habits among students. These include the high frequency of snacking (between meals) [7], the tendency to eat sugar-rich foods [8], and heavy alcohol consumption [9]. In Cameroon, few studies have been conducted among university students to assess the level of malnutrition. To date, Kana et al's study [10] at the University of Douala is the only one reported to our knowledge. However, the implementation of strategies to prevent associated comorbidities in adulthood requires intervention starting at a young age. Hence, this study aims not only at providing concrete figures on the situation of malnutrition among students of the University of Maroua, but also in describing their eating habits and food consumption according to their place of residence and identifying the specific risk factors associated with malnutrition in this young population living in one of the most food-insecure region of the country (the Far North with 33.7% of food-insecure households) [11].

## 2. Methodology

### 2.1. Description of the Place and Period of Study

A cross-sectional, descriptive, and analytical study was carried out from 14 January to 28 February 2018 at the University of Maroua in the Far North Region of Cameroon. Students were recruited at the Faculty of Human and Social Sciences (FHSS); the Faculty of Science (FS); the Faculty of Economics and Management Science (FEMS); the Faculty of Law and Political Sciences (FLPS); the National Polytechnic High School (NPHS), and the Higher Teachers' Training College (HTTC) of the University of Maroua. The recruitment of students was done at Kongola (HTTC) and Ouro-Chede (FHSS, FEMS, FLPS, and NPHS) campuses.

### 2.2. Study Population, Sample Size, and Eligible Criteria

Students of both sexes, volunteers, Cameroonians, and Chadians aged between 17 and 35 years and apparently healthy were eligible for the survey.

The sample size of the study was calculated using the Magnani formula [12].

(1) $$\begin{array}{c} n=\frac{t^{2}\times p\left(1-p\right)}{m^{2}}, \end{array}$$

where *n* is the required sample size, *t* is the confidence level at 95% (standard value of 1.96), *p* is the estimated prevalence of overweight in the Cameroonian adult population, which was 26% [13], and *m* is the margin of error at 5% (standard value of 0.05).

(2) $$\begin{array}{c} n=\frac{1.96^{2}\times 0.26\left(1-0.26\right)}{0.05^{2}}=295. \end{array}$$

Therefore, the total sample size required for this study was 295 students. A total of 348 volunteers were recruited during the survey, and after cleaning the database, 330 of them had their questionnaire fully completed and were enrolled in this study.

### 2.3. Ethical Considerations

The study protocol was approved by the National Ethics Committee N 2014/08/488/EC/CNERSH and was conducted in strict compliance with the physical, moral, and psychological integrity of all participants.

### 2.4. Anthropometric Measurements and Assessment of Nutritional Status

Weight was measured using a calibrated electronic scale (TANITA), with a range of 25 to 150 Kg and an accuracy of 0.5 Kg, while a stadiometer was used to measure the standing height of participants to the nearest centimeter. Measurements were done by a well-trained staff. Body mass index (BMI) was computed using these two parameters. Thus, any BMI value between 25 and 29.9 kg/m^2^ was considered overweight and those with a BMI > 30 kg/m^2^ were classified as obese. Participants with a BMI ≤of 18.5 kg/m^2^ were considered underweight [14].

Waist circumference (WC) was taken at the mid-point between the bottom rib and the hip bone, without restrictive garments, while hip circumference (HC) was measured at the point of greatest circumference around the hips using a flexible non-expandable tape measure to the nearest 0.1 cm. Waist-to-hip ratio (WHR) was taken as the proportion between WC and HC cut-off.

### 2.5. Questionnaire and Interview

Each participant was invited to a face-to-face interview with a well-trained surveyor. The surveyors were trained on the nutritional survey. A questionnaire inspired from the WHO STEPwise approach to NCD risk factor surveillance (STEPS) instrument was developed. This permitted to have sociodemographic information (age, gender, country of origin, level of education, place of residence, marital status, eating habits, food security, and consumption).

### 2.6. Assessment of Eating Habits: Frequency of Consumption of the Various Food Groups and Food Intake

Eating habits were assessed through a face-to-face interview with each participant using a two-part questionnaire:

- The first part was a 7-day recall food frequency questionnaire that permits to evaluate the frequency of consumption of various food groups (protein, carbohydrates, dairy products, fruits, vegetables, etc.) using a list of foods that are usually consumed by the population of the Far North Region of the country. Each student was invited to give the frequency of consumption of the food consumed over the past 7 days. Subsequently, the data obtained allowed to classify each food group into 3 categories: 0–1 time per week as low consumption, 2–4 times per week as moderate consumption, and 5–7 times per week as high consumption.
- The second part of the questionnaire was a 24-hour recall of dietary consumption (day/week). Each participant was asked to mention everything that he/she had eaten the day before the survey (from waking up to sleeping in night), specifying the quantities in terms of local household measurements. Local household utensils were presented to students to facilitate the description of portions of food consumed. Subsequently, these 24-hour recalls were analyzed, and a proxy of macronutrients and energy intake was estimated using the West African food composition table in the absence of the local one.

Energy intake was compared to the Estimated Average Requirements (EARs) and macronutrient intake to the Reference Nutrient Intake (RNI). In addition, Energy percent (E%) from protein, carbohydrate, and fat from total calories consumed were assessed and compared to the EARs [15].

### 2.7. The Dietary Diversity Score (DDS)

A dietary diversity score (DDS) has been considered as an indicator of diet quality associated inversely with the risk of chronic diseases. It is also an approximate measurement of the nutritional adequacy of the diet at the individual level [16]. DDS was computed by counting the number of food groups (10) (Table 1) consumed by the respondent over a 24-hour period and recorded on the 24-h recall of dietary consumption. A rating of “1” was assigned if the group was present in the 24-hour recall and “0” in the absence of this group. The DDS was computed as a total number of group of foods consumed to a maximum of 10. Participants were grouped into three categories according to their DDS: poor-diversified diet (DDS between 0 and 3), moderate-diversified diet (DDS of 4–5), and diversified diet (DDS of 6–10) [17, 18].

### 2.8. Food Consumption Score (FCS)

The food consumption score (FCS) is a composite score based on the diversity of the diet, the frequency of food consumption, and the importance of the nutrients contained in the different food groups. Eight groups of food were considered (Table 2) and a table was created indicating the frequency of consumption of each group of food. Each food group had a corresponding quality weighing factor that reflected its energy value. This weighing factor was based on the density of nutrients contained in the food consumed. The frequency of consumption of each weighed and summed food group constituted the FCS as described by the following formula:

(3) $$\begin{array}{c} \text{FCS}=a_{1}X_{1}+a_{2}X_{2}+a_{3}X_{3}+a_{4}X_{4}+a_{5}X_{5}+a_{6}X_{6}+a_{7}X_{7}+a_{8}X_{8}, \end{array}$$

where *X* is the number of days of consumption of each food group during the past 7 days and *a* is the weight assigned to the food group.

After computing, the FCS was reported on a scale with standard thresholds used to determine the three categories of food consumption: FCS from 0 to 28 refers to low food consumption, FCS between 28.5 and 42 referred to limited food consumption, and FCS >42 was considered as acceptable food consumption [19].

### 2.9. Food Insecurity

Food insecurity was assessed using the simple method recommended by Swindale and Bilinsky [20]. The questionnaire identifies the problems of access to food and lack of food. For each item, the response was rated from 0 to 1 or 3 depending on the frequency or severity of the situation. The aggregation of response scores determines a food insecurity score (FIS). Participants were grouped into three categories according to their FIS: FIS of 0 to 4 correspond to mild food insecurity, FIS between 5 and 7 as moderate food insecurity, and FIS of 8 and more corresponded to high food insecurity.

### 2.10. Data Entry, Processing, and Statistical Analysis

The data were entered into an Excel worksheet 2010 and transferred to Statistical Package for Social Sciences (SPSS) software version 20.0 for Windows for statistical analysis. The results were expressed as means ± standard deviation (SD) and frequency (%). Descriptive statistics made it possible to describe the distribution of each variable under study to determine its characteristics (frequency, mean, SD). Pearson's chi-square test was used to compare the proportions between categorical variables. The Student's *t*-test was used to compare means between two groups of continuous variables. The significance threshold was set at *p* < 0.05.

## 3. Results

The study population consisted of 330 students, 63.6% Cameroonians and 36.4% Chadians. Among these, 81.6% were recruited at the undergraduate and 18.5% at the graduate level. Concerning the school attended, 28.2% of the study participants were in training schools (Higher Teachers' Training College and National Polytechnic High School) while 71.8% were in the faculties (Faculty of Science, Faculty of Human and Social Sciences, and Faculty of Law and Political Sciences). Furthermore, 70.3% lived alone not with their families (Table 3).

Results of Table 5 revealed that 13% of students were overweight, 6.1% were underweight, and all age groups were affected.

### 3.1. Assessment of Food Habits among Students

Cereals (maize, rice, bread, pasta, wheat flour, sorghum, millet, etc) were the main carbohydrate-rich foods consumed rather than tubers (potatoes, yams, cassava, etc.) (Table 6). Concerning protein-rich foods and dairy products, the results show a significant proportion of participants with a low frequency of protein consumption (at most once a week) regardless of the source. The least consumed ones were poultry (95.5% of study participants consumed it at most once a month) and dairy products. A very low frequency of fruit consumption was also observed, only 12.1% (for fleshy fruits) and 7.3% (for dried fruits) of participants consumed them 5 to 7 times a week and men were the most concerned. Vegetables were grouped into three categories: fresh and dried green leaves (*Hibiscus sabdariffa, Moringa oleifera, Adansonia digitata, Amaranthus hybridus, Vernonia amygdalina, Manihot esculenta, Abelmoschus esculentus*, etc) and other vegetables (carrot, tomato, cucumber, cabbage, etc). As a matter of fact, 17.9% (fresh green leaves), 17.6% (dry green leaves), and 22.1% of students consumed them 5 to 7 times a week. Regardless of alcoholic beverages (traditional and industrial beers), hot drinks (tea, coffee, chocolate, etc), and sweets (candies, sweet snacks, sweet bread, sweet biscuits, cakes, chocolate, etc), it was noted that 38.8% of the respondents were alcohol consumers and mostly men. The high sugar intake was assessed through the frequency of consumption of hot drinks and sweets, and results indicated an increased proportion of participants with moderate to high consumption of sweets (Table 6). When considering the place of residence, no difference was observed among students except for the consumption of red meat, eggs, fresh green vegetables, and other vegetables with a significantly high proportion of students living alone presenting a low frequency of consumption compared to those living with their family (*p* < 0.05). According to gender, women exhibited a low frequency of consumption of pulses, dried fruits, and vegetables as well as traditional meals, while men were characterized by a low frequency of consumption of red meat, eggs, dairy products, and fresh fruits (*p* < 0.05).

### 3.2. Evaluation of Food Consumption, Food Diversity, and Food Insecurity in the Study Population

The mean DDS, FCS, and FIS were 4.34 ± 1.14, 61.99 ± 20.57, and 2.32 ± 2.33, respectively, among students with no significant difference between gender or place of residence for these scores (Table 7). Classification of students according to their scores revealed that 21% (*n* = 69) of them had a poorly diversified diet, only 6% (*n* = 18) had a poor food consumption score, and only 2% (*n* = 7) of them were highly food insecure. However, no statistical difference was found between gender and place of residence.

### 3.3. Quantitative Assessment of Students' Macronutrients and Energy Intake

The 24-h recall diary was used to quantify macronutrients and energy intake (Table 8). The daily macronutrient intake of the students was within the recommended reference value range, except for carbohydrates whose value was above the recommended range. When assessing the energy intake of each macronutrient, carbohydrates represented 62.69 ± 13.84% of the daily energy intake. It was also noted that only 32.2% of students had an energy intake that meets the recommendations (Table 8).

### 3.4. Interaction between Nutritional Status and Socioeconomic and Nutritional Factors

The identification of factors that could affect the nutritional status of students revealed that being married (13.4%) (*p* < 0.05) was associated with a high frequency of being overweight (14.7%), whereas living with family (11.5%) (*p* < 0.05) was associated with a high frequency of underweight as shown in Table 9.

## 4. Discussion

This study, which aims at evaluating malnutrition among students of the University of Maroua, reveals that both forms of malnutrition coexisted among these students: 13% of them were overweight (12.1% for overweight and 0.9% for obesity) and 6.1% were underweight (Table 5). The prevalence of overnutrition was lower as compared to the 19.4% (for overweight) and 4.1% (for obesity) obtained by Madengué et al. [21] among the students of the University of Douala. This results were also lower than that of Niba et al. [22] among the students of the University of Bamenda in Cameroon (24.6% overweight and 2.2% obese). The difference could be attributed to the degree of urbanization of the cities hosting these universities—Douala city is a metropolis and Bamenda city is more urbanized than Maroua city. Indeed, it has been demonstrated that urbanization contributes significantly to the explosion of obesity and its complications [23]. Regarding underweight, our results (6.1%) were higher than the 4.6% reported by Madengué et al. [21]; this can be due to the geographic localization of the University of Maroua which is located in one of the most food-insecure regions of the country, where populations are more exposed to undernutrition [11]. Concerning gender, it was noted that women were the most affected by both being underweight and overweight even if men had a higher WC and WHR (Table 5). The female predominance of overweight or obesity could be explained by the physiology of the girl, which is marked by hormonal development promoting fat mass gain, while in the young boy, muscle development (lean mass) is more pronounced.

Qualitative analysis of food consumption revealed a significant proportion of participants with a low frequency of protein-rich food consumption regardless of the source (Table 6), which are foods known as high energy and protein content with low fat [24]. Our results differ from the observations made by Niba et al. [22] about meat and fish consumption. They found that 30.7 to 32.9% of students consumed meat and fish at most once a week while 23.1 to 27.9% of them consumed it 5–7 times a week in Bamenda. This was also true for egg consumption, which was higher in their population than ours. The geographical location of Maroua city (Northern Sudano-Sahelian zone) could play a key role in the availability of certain protein-rich foods and therefore limit their consumption [25]. Concerning carbohydrate sources, it was noted that cereals were highly consumed compared to tubers, and this could be justified by the fact that the University of Maroua is located in the Far North region of Cameroon, which is an area of high cereal production and therefore more available [26] than tubers which are imported from the southern part of the country, hence reducing their availability and increasing their cost in the local market [25]. The low fruit consumption observed among these students of the University of Maroua (Table 6) could be a consequence of their limited availability in the local market, their high price, and their lack of financial resources of students. The gender difference in fruit consumption (women consumed more than men) observed in this study (Table 6) was also noted by Azagba's among Canadians [27]. Blanck et al. [28] made the same observations among young people in the United States and justified this by the fact that women would have a better level of nutritional knowledge and were more concerned about their health than men. For vegetables, men consumed more vegetables (dried ones as well as dried fruits) than women, this observation is contrary to many studies which had always reported that men had the lowest frequency of consumption because they could not cook food themselves [29] and preferred ready-to-eat foods. When considering the place of residence of the participants, living with the family was associated with a high proportion of participants eating vegetables, red meat, and eggs about 5–7 times per week compared to those living alone, confirming the fact that living in university milieu is associated with some food habits changes [30]. Ansari et al. [29] also noted that students living with their parents consumed more fruit, vegetables, and meat than those who resided outside of their family home in a multicountry study. Indeed, as students leave home and start independent living, good dietary habits decline. This might be because those living with their parents do not have to pay for food and therefore do not suffer from financial limitations in this respect. In addition, meals containing vegetables and other healthy food items might be prepared for them and thus more healthy food is available for them [31].

The analysis of food consumption data (Table 7) shows that 81% of students had an acceptable food intake versus 19% who had an unsatisfactory diet (low/limited food intake). These results were closer to those obtained by the World Food Program (WFP) [25] among populations of the Far North Region of Cameroon, where 17.9% had an unsatisfactory diet and 82.1% with an acceptable food intake. The evaluation of the food diversity (Table 7) showed that 21% (*n* = 69) of the students surveyed had a poorly diversified diet. These results were lower than Ayouba's [32] observations in urban areas of Niger, where 38.46% of households had a poor diversity diet and 0% had a highly diversified diet. The lack of financial resources, limited diet, availability of some groups of foods, high cost, and attachment to traditional meals (monotonous) may contribute to the low diversity diet of this population [18, 33]. But, at the level of food security, the majority of students surveyed (82%; *n* = 272) fell in the food security category; meanwhile, only 2% (*n* = 7) of them were highly food insecure (Table 7). These results were also confirmed by the high proportion of students with acceptable food consumption (81%, *n* = 268) observed in our study population (Table 7). These results were nearest to Michael et al.'s [34] observations, who found that 1% of students at the University of Witwatersrand in South Africa were severely food insecure and 6% were moderately food insecure.

The daily macronutrient intake of the students was within the recommended reference values, except for carbohydrates which exceeded (Table 8), this implies an unbalanced diet [35, 36]. These results can be explained by the high consumption of cereals and hot drinks/sweetened foods by students (Table 6). Similar observations have been made by Kana et al. [10] among students of the University of Douala. In addition to high carbohydrate intake, the authors found a very low protein intake. It should be noted that no significant difference was observed among genders. This suggests that the high prevalence of overweight and obesity among women is not due to energy intake but probably a consequence of a low level of physical activities that generally characterize women [10]. Concerning the energy intake from each macronutrient, the results indicated that carbohydrates constituted 62.69 ± 13.84% of daily energy intake, which is above the recommended range value (50–55%) [13]. This is a characteristic of a lower-income population diet that differs from a higher-income population where protein and fat intake are usually exceeded [37, 38]. In terms of energy, the results of this study were different from those of Kana et al. [10] among Douala University students, where all participants had an energy intake that meets the recommendations. The difference could be explained by limited availability, with food inaccessibility leading to undernutrition among Maroua University students [39]. Among the students who participated in this survey, only 32.2% (*n* = 107) of them had an energy intake that meets recommendations. On the other hand, 67.7% had a daily energy intake below the recommendations, which could partly justify the significant prevalence of underweight observed in the study (Tables 5 and 8).

When looking at factors that can lead to malnutrition in this study population, it appears that marital status “married” was associated with the high prevalence of overweight (Table 9). This result could be explained by the fact that married students have socioeconomic stability which allows them to eat properly, be food secure [40], and therefore leads to weight gain [36]. Regardless of the place of residence, the high prevalence of underweight was noted among students who lived with family (Table 9). This finding could be partly due to poverty; in the specific and cultural context of the populations of the Far North region of Cameroon, many families always eat their meals together on the same plate so the portions ingested by each can be reduced, hence the observed underweight.

In conclusion, the double burden of malnutrition is present among students at the University of Maroua; most of them had a poorly diversified diet characterized by carbohydrate energy intake above the recommended range. Finally, marital status was associated with being overweight, meanwhile living with their family increased the risk of undernutrition. Nutritional education for these young people would help them make a better choice of food and adopt good practices which can help to prevent nutritional related complications once they attend active life.

## Figures and Tables

**Table 1:** Group of food used for the DDS.

Food groups	Food
(1) Cereals	Maize, rice, bread, pasta, wheat flour, wheat couscous, millet
(2) Root and tubers	Manioc, yam, plantain banana, sweet potato, potato
(3) Pulses/nuts	Beans, soybeans, peanuts, cola nuts, coconuts, palm nuts, sesame seeds
(4) Meat and poultry	Beef, mutton, goat, pork, rabbit, chicken, turkey, guinea fowl
(5) Fish and seafood	Fish, crabs, shrimps, snails
(6) Eggs	Fried and boiled eggs
(7) Milk and milk products	Whole milk powder, sweetened condensed milk, curdled milk, semi-skimmed milk, yogurt, local and imported cheeses
(8) Fruits	Mangoes, pineapples, bananas, avocados, guavas, melons, oranges, mandarins
(9) Vegetables	Cabbage, lettuce, tomato, carrots, okra, cucumber, spinach, green beans
(10) Oil and fat	Vegetable oils, margarine, butter, mayonnaise

**Table 2:** Group of food used for FCS.

Food items	Food group	Weight (a)
Maize, rice, sorghum, millet, bread, and other cereals	Cereals and tubers	2
Cassava, potatoes, and sweet potatoes		
Beans, peas, groundnuts, and cashew nuts	Pulses	3
Vegetables, relish, and leaves	Vegetables	1
Fruits	Fruits	1
Beef, goat, poultry, pork, and fish	Meat and fish	4
Milk, yogurt, and other dairy products	Milk	4
Sugar and sugar products	Sugar	0.5
Oils, fats, and butter	Oil	0.5

**Table 3:** Overall description of the study population.

Parameters	Variables	Frequency	Percentages (%)
Nationality	Cameroonian	210	63.6
	Chadian	120	36.4

Gender	Female	89	27.0
	Male	241	73.0

Level of education	Undergraduate	269	81.6
	Graduate	61	18.5

Establishments attended	Higher Teachers' Training College	64	19.4
	Faculty of Science	78	23.6
	Faculty of Human and Social Sciences	124	37.6
	Faculty of Law and Political Sciences	17	5.2
	Faculty of Economics and Management Science	18	55
	National Polytechnic High School	29	8.8

Place of residence	Living with family	109	33.0
	Living alone	221	67.0

Marital status	Married	34	10.3
	Single	296	89.7

The mean age of the participants was 23.95 ± 3.67 years and BMI was 22.19 ± 2.78 kg/m2. Furthermore, age and waist-to-hip ratio were significantly higher among men while women exhibited a high BMI (*p* < 0.05) (Table 4).

**Table 4:** Anthropometric characteristics of the study population.

Variables	Overall	Women	Men	*P*-value	*Living alone*	*Living with family*	*P*-value
Age (years)	23.95 ± 3.67	23.21 ± 3.46	24.22 ± 3.71	0.027	24.02 ± 3.74	23.81 ± 3.53	0.624
BMI (kg/m^2^)	22.19 ± 2.78	23.06 ± 3.60	21.87 ± 2.33	0.001	22.27 ± 2.61	22.04 ± 3.09	0.480
WC (cm)	76.09 ± 7.53	74.81 ± 9.62	76.57 ± 6.55	0.060	76.99 ± 7.41	74.27 ± 7.47	0.002
HC (cm)	92.26 ± 7.63	93.46 ± 10.66	91.82 ± 6.12	0.082	92.85 ± 7.47	91.07 ± 7.86	0.046
WHR	0.83 ± 0.07	0.80 ± 0.08	0.83 ± 0.06	0.0001	0.83 ± 0.07	0.82 ± 0.06	0.064

BMI, body mass index; WC, waist circumference; HC, hip circumference; WHR waist-to-hip ratio.

**Table 5:** Prevalence of malnutrition among students of the University of Maroua.

	Undernutrition (BMI < 18.5 kg/m^2^) *n* (%)	Overweight (25 kg/m^2^ ≤ BMI ≥ 29.9 kg/m^2^) *n* (%)	Obesity (BMI ≥ 30 kg/m^2^) *n* (%)
Overall	20 (6.1)	40 (12.1)	03 (0.9)
Women	06 (6.7)	20 (22.5)	02 (2.2)
Men	14 (5.8)	20 (8.3)	01 (0.4)
17–25 years	18 (7.5)	24 (10)	01 (0.4)
26–30 years	00 (0)	11 (17.5)	00 (0)
31–35 years	02 (7.7)	05 (19.2)	02 (7.7)

**Table 6:** Description of food habits of the students.

Food groups	Frequency of consumption	Overall N = 330 n (%)	Gender			Place of residence		
			Men *n* (%)	Women *n* (%)	*P*-value	Alone *n* (%)	With family *n* (%)	*P*-value
Cereals	Low	89 (26.8)	59 (24.6)	29 (32.6)	0.335	58 (26.2)	30 (27.8)	0.681
	Moderate	105 (31.9)	78 (32.5)	27 (30.3)		74 (33.5)	31 (28.7)	
	High	136 (41.3)	103 (42.9)	33 (37.1)		89 (40.3)	47 (43.5)	

Tubers	Low	242 (73.4)	179 (74.3)	63 (70.7)	0.433	167 (75.6)	75 (68.8)	0.290
	Moderate	64 (19.4)	43 (17.8)	21 (23.6)		41 (18.6)	23 (21.1)	
	High	24 (7.3)	19 (7.9)	5 (5.7)		13 (5.9)	11 (10.1)	

Pulses	Low	226 (68.5)	155 (64.3)	71 (79.8)	0.007	150 (67.9)	79 (69.7)	0.857
	Moderate	60 (18.2)	46 (19.1)	14 (15.7)		42 (19)	18 (16.5)	
	High	44 (13.3)	40 (16.6)	4 (4.5)		29 (13.1)	15 (13.8)	

Fish	Low	188 (56.9)	133 (55.4)	54 (60.7)	0.215	124 (56.1)	63 (58.3)	0.889
	Moderate	94 (28.6)	68 (27.9)	27 (30.3)		65 (29.4)	29 (26.9)	
	High	48 (14.6)	40 (16.7)	8 (9.0)		32 (14.5)	16 (14.8)	

Poultry	Low	315 (95.5)	228 (94.6)	87 (97.7)	0.220	208 (94.1)	107 (98.2)	0.251
	Moderate	7 (2.1)	5 (2.1)	2 (2.3)		6 (2.7)	1 (0.9)	
	High	8 (2.4)	8 (3.3)	0 (0)		7 (3.2)	1 (0.9)	

Red meat	Low	198 (60)	159 (67)	39 (43.8)	0.001	148 (67)	50 (45.9)	0.001
	Moderate	88 (26.7)	54 (22.4)	34 (38.2)		48 (21.7)	40 (36.7)	
	High	44 (13.3)	28 (11.6)	16 (18.0)		25 (11.3)	19 (17.4)	

Eggs	Low	264 (80)	205 (85)	59 (66.3)	0.001	175 (79.2)	89 (81.7)	0.048
	Moderate	44 (13.3)	23 (9.5)	21 (23.6)		35 (15.8)	9 (8.3)	
	High	22 (6.7)	13 (5.5)	9 (10.1)		11 (5)	11 (10.5)	

Dairy products	Low	134 (70.9)	179 (74.3)	55 (61.8)	0.023	158 (71.5)	76 (69.7)	0.946
	Moderate	61 (18.5)	36 (14.9)	25 (28.1)		40 (18.1)	21 (19.3)	
	High	35 (10.6)	26 (10.8)	9 (10.1)		23 (10.4)	12 (11)	

Fresh fruits	Low	211 (63.9)	205 (68.5)	46 (51.7)	0.006	147 (66.5)	64 (58.7)	0.260
	Moderate	79 (23.9)	23 (19.5)	32 (36.0)		47 (21.3)	32 (29.4)	
	High	40 (12.1)	13 (12.0)	11 (12.3)		27 (12.2)	13 (11.9)	

Dried fruits	Low	280 (84.9)	197 (81.8)	83 (93.3)	0.017	189 (85.5)	91 (83.5)	0.826
	Moderate	26 (7.9)	21 (8.7)	5 (5.6)		16 (7.2)	10 (9.2)	
	High	24 (7.3)	23 (9.5)	1 (1.1)		16 (7.2)	8 (7.3)	

Dried green leaves	Low	188 (56.9)	125 (51.9)	63 (70.7)	0.003	131 (59.3)	57 (52.3)	0.148
	Moderate	84 (25.5)	66 (27.0)	19 (21.3)		49 (22.2)	35 (32.1)	
	High	58 (17.6)	50 (21.2)	7 (8)		41 (18.6)	17 (15.6)	

Fresh green leaves	Low	182 (55.1)	134 (55.6)	48 (54)	0.250	138 (62.4)	44 (40.4)	0.001
	Moderate	89 (27.0)	60 (24.9)	29 (32.6)		51 (23.1)	38 (34.9)	
	High	59 (17.9)	47 (19.5)	12 (13.4)		32 (14.5)	27 (24.8)	

Others vegetables	Low	160 (48.5)	125 (51.9)	35 (39.3)	0.129	118 (53.4)	42 (38.5)	0.023
	Moderate	97 (29.4)	66 (27.4)	31 (34.8)		62 (28.1)	35 (32.1)	
	High	73 (22.1)	50 (20.7)	23 (25.8)		41 (18.6)	32 (29.4)	

Traditional meals	Low	185 (59.5)	127 (55.9)	58 (69.0)	0.015	128 (60.7)	57 (57)	0.825
	Moderate	68 (21.9)	49 (21.6)	19 (22.6)		45 (21.3)	23 (23)	
	High	58 (18.6)	51 (22.5)	7 (8.3)		38 (18)	20 (20)	

Hot drinks	Low	171 (51.8)	117 (48.6)	54 (60.6)	0.146	124 (56.1)	47 (43.1)	0.083
	Moderate	71 (21.5)	55 (22.8)	16 (18.0)		44 (19.9)	27 (24.8)	
	High	88 (26.7)	69 (28.6)	19 (21.4)		53 (24)	35 (32.1)	

Sweet foods	Low	167 (50.6)	126 (52.3)	41 (46.1)	0.574	115 (52)	52 (47.7)	0.638
	Moderate	88 (26.7)	63 (26.1)	25 (28.1)		59 (26.7)	29 (26.6)	
	High	75 (22.7)	52 (21.6)	23 (25.8)		47 (21.3)	28 (25.7)	

Alcoholic beverages	No	225 (68.2)	158 (65.6)	67 (75.3)	0.092	75 (68.8)	150 (67.9)	0.864
	Yes	105 (38.8)	83 (34.4)	22 (24.7)		71 (32.1)	34 (31.2)	

Low, 0–1 time per week; moderate, 2–4 times per week; high, 5–7 times per week.

**Table 7:** Dietary diversity, food consumption, and food insecurity among study participants.

		Overall	Gender			Place of residence		
			Women	Men	*P*-value	Living alone	Living with family	*P*-value
Results expressed as means ± SD								
Dietary diversity score (DDS)		4.34 ± 1.14	4.37 ± 1.18	4.32 ± 1.13	0.769	4.33 ± 1.15	4.36 ± 1.13	0.872
Food consumption score (FCS)		61.99 ± 20.57	62.56 ± 18.69	6.78 ± 21.25	0.761	62.28 ± 20.69	61.42 ± 20.39	0.721
Food insecurity score (FIS)		2.32 ± 2.33	2.51 ± 2.16	2.25 ± 2.39	0.376	2.32 ± 2.23	2.32 ± 2.53	0.987

*Results expressed as % (N)*								
Dietary diversity	Poor	21 (69)	20.2 (18)	21.1 (51)	0.983	21 (46)	20.6 (22)	0.599
	Moderate	66 (218)	66.3 (59)	65.8 (158)		67.1 (147)	63.6 (68)	
	Diversified	13 (43)	13.5 (12)	13.1 (32)		11.9 (26)	15.9 (17)	
Food consumption	Poor	6 (18)	3.4 (3)	6.2 (15)	0.598	5.4 (12)	5.5 (6)	0.986
	Borderline	13 (44)	13.5 (12)	13.3 (32)		13.1 (29)	13.8 (15)	
	Acceptable	81 (268)	83.1 (74)	80.5 (194)		81.4 (180)	80.7 (88)	
Food insecurity	Low	82 (272)	83.1 (74)	82.2 (198)	0.746	81.4 (180)	84.4 (92)	0.199
	Mild	16 (51)	15.7 (14)	15.4 (37)		17.2 (38)	11.9 (13)	
	High	2 (7)	1.1 (01)	2.5 (6)		1.4 (3)	3.7 (4)	

**Table 8:** Macronutrients and energy intake of the study participants.

	Recommended daily intake	Overall	Men	Women	Sig.
Macronutrients intake					
Proteins (g)	54–90 g	69.54 ± 50.70	72.53 ± 56.34	60.58 ± 25.90	0.192
Fats (g)	54–100 g	57.15 ± 34.61	57.41 ± 33.72	56.36 ± 37.58	0.868
Carbohydrates (g)	200–300 g	339.80 ± 156.80	346.53 ± 163.84	319.60 ± 133.23	0.343

*Energy intake*					
Energy (kcal)	2200–2700 Kcal	2151.78 ± 835.02	2192.99 ± 869.31	2028.14 ± 718.02	0.275
Adequate energy intake % (n)	Yes	32.2 (107)	30.1 (73)	39 (35)	0.289
	No	67.7 (223)	69.9 (168)	61 (54)	

*Percentage of macronutrients energy intakes*					
% Proteins	10–15%	12.72 ± 6.04	12.84 ± 6.44	12.38 ± 4.7	0.679
% Fats	30–35%	24.60 ± 13.07	24.41 ± 13.01	25.12 ± 13.40	0.764
% Carbohydrates	50–55%	62.69 ± 13.84	62.75 ± 14.01	62.49 ± 13.48	0.918

**Table 9:** Assessment of factors influencing the nutritional status of students of the University of Maroua.

		Underweight		Overweight	
		% (*N*)	*P*-value	% (*N*)	*P*-value
Food diversity	Poor	8.8 (6)	0.283	14.7 (10)	0.826
	Average	4.7 (10)		14.0 (27)	
	High	9.3 (4)		11.6 (5)	

Food consumption	Poor	0 (0)	0.187	27.8 (5)	0.192
	Borderline	11.4 (5)		13.6 (6)	
	Acceptable	5.6 (15)		11.9 (32)	

Food insecurity	Low	6.6 (18)	0.574	13.6 (37)	0.721
	Moderate	3.9 (2)		9.8 (5)	
	High	0 (0)		14.3 (1)	

Energy requirement	Satisfactory	1.9 (1)	0.070	17 (9)	0.711
	Unsatisfactory	9.9 (11)		13.5 (15)	

Place of residence	With family	10.1 (11)	0.028	13.8 (15)	0.619
	Alone	4.1 (9)		12.7 (28)	

Level of education	Undergraduate	5.6 (15)	0.381	12.3 (33)	0.340
	Graduate	8.2 (5)		16.4 (10)	

Holding a paid job	Yes	3.7 (2)	0.424	13 (7)	0.925
	No	6.5 (18)		13 (36)	

Marital status	Married	5.9 (2)	0.879	23.5 (8)	0.005
	Single	6.1 (18)		11.8 (35)	

## Data Availability

The data used to support the findings of the study can be obtained from the corresponding author upon request.

## Additional Points

The assessment of micronutrient intake would have given more information about malnutrition characteristics among these students.
